# Neurological Complications of Type 2 Diabetes Mellitus: A Duration-Based Comparative Study at a Secondary Care Hospital in Karachi, Pakistan

**DOI:** 10.7759/cureus.88562

**Published:** 2025-07-23

**Authors:** Muhammad Adil Ramzan, Hamda Rehman, Bhavesh Kumar, Abdul Ghafoor, Neeta Maheshwary, Waseem Asif, Adnan Anwar, Atif A Hashmi

**Affiliations:** 1 Medicine, Abbasi Shaheed Hospital, Karachi, PAK; 2 Internal Medicine, United Medical and Dental College, Karachi, PAK; 3 Internal Medicine, Ziauddin University, Karachi, PAK; 4 Pharmacology and Therapeutics, Ziauddin University, Karachi, PAK; 5 Physiology, Hamdard College of Medicine and Dentistry, Karachi, PAK; 6 Internal Medicine, Sindh Government Hospital, Karachi, PAK; 7 Pathology, Liaquat National Hospital and Medical College, Karachi, PAK

**Keywords:** duration, muscular pain, neurological symptoms, tingling, type 2 diabetes mellitus

## Abstract

Objective

Type 2 diabetes mellitus (T2DM) often leads to neurological complications that tend to increase in severity with disease duration. This study compared the prevalence of neurological symptoms in individuals with T2DM with respect to the duration of disease.

Methodology

This cross-sectional study was carried out over a six-month period at secondary care hospitals. A total of 420 patients with T2DM, aged between 40 and 65 years, were categorized into three equal groups of 140 based on duration of diabetes. Group A included newly diagnosed patients (less than or equal to one year), Group B comprised individuals with one to five years of diabetes, and Group C consisted of those with a duration exceeding five years. Clinical evaluations focused on glycemic control (glycated hemoglobin (HbA1c) and postprandial glucose) and neurological complications involving peripheral, autonomic, and central nervous systems. Data were analyzed using IBM SPSS Statistics for Windows, Version 20 (Released 2012; IBM Corp., Armonk, New York, United States), with chi-square and Mann-Whitney tests applied, with p-values <0.05 taken as statistically significant.

Results

This study’s findings showed that Group A (less than or equal to one year) had significantly higher mean age, weight, BMI, respiratory rate, heart rate, and random blood sugar levels compared to Groups B and C. Gender distribution varied significantly, with all males in Group A and more females in Group B. Dyslipidemia and smoking history were significantly associated with diabetes duration (p < 0.001), while hypertension was not. Neuropathy symptoms, especially tingling and numbness, were more common in Group C, while autonomic symptoms like fatigue and irritability were highest in Group B. Socioeconomic status and type of therapy also showed significant differences across groups (p < 0.05).

Conclusion

This study concluded that peripheral neuropathy symptoms were more common in patients with longer diabetes duration, indicating progressive nerve damage over time. Some symptoms, such as burning pain and fatigue, appeared earlier, suggesting early metabolic changes. Autonomic symptoms also varied significantly, highlighting the complex progression of neurological involvement in T2DM.

## Introduction

Type 2 diabetes mellitus (T2DM) is widely recognized as a major public health issue, significantly affecting both individuals' well-being and healthcare costs [1]. It impairs functional ability and quality of life, contributing to high levels of illness and early death [2]. Alarming reports indicate that over one-third of diabetes-related fatalities occur in individuals younger than 60 [3]. Recent data shows a continued global rise in T2DM cases. As of 2021, 537 million adults (10.5%) aged 20-79 are affected, an increase of 74 million since 2019, marking a 16% surge [4]. Projections by the International Diabetes Federation (IDF) estimate this number will reach 643 million by 2030 (11.3%) and 783 million by 2045 (12.2%), reflecting a 46% rise, more than double the expected global population growth of 20%. By then, nearly one in eight adults may have diabetes [4,5].

Approximately 10% of the global population is affected by diabetes, with T2DM making up about 90% of these cases [6,7]. The condition is influenced by a wide range of risk factors and is linked to multiple complications [8-10]. The brain is highly sensitive to glucose levels, which play a crucial role in maintaining neurons, supporting neurogenesis, regulating neurotransmitters, and enabling synaptic plasticity [11]. It is estimated that 30% to 50% of individuals with diabetes may develop neuropathy [12].

Diabetic peripheral neuropathy (DPN) is a common and serious complication of T2DM, with its prevalence varying between 20% and 80% depending on the region [13]. Painful diabetic peripheral neuropathy (PDPN), a form of DPN marked primarily by pain [14], differs from its painless counterpart as it is often associated with disrupted sleep, decreased productivity, lower quality of life, mental health issues like anxiety and depression, and increased medical expenses [15].

Symptom severity in diabetes varies based on its type and duration. Early-stage T2DM may be asymptomatic, while individuals, especially children, with severe hyperglycemia due to insulin deficiency can present with classic symptoms like polyuria, polydipsia, polyphagia, weight loss, and blurred vision. Without proper management, diabetes may lead to serious complications such as diabetic ketoacidosis or, less commonly, non-ketotic hyperosmolar syndrome, potentially resulting in confusion, coma, or death [16].

Timely diagnosis and identification of individuals at risk are essential for implementing strategies to prevent complications related to DPN [17,18]. DPN symptoms range from discomfort and loss of protective sensation, typically starting in the toes and progressing in a stocking-like pattern, to more severe complications. “Positive symptoms” drive patients to seek care, while “negative symptoms” involve unnoticed loss of sensation, leading to unrecognized injuries. Without early detection and preventive measures like proper foot care and footwear, patients face a heightened risk of foot ulcers and potential lower limb amputation [19]. For this reason, the American Diabetes Association (ADA) advises that all individuals with diabetes be screened for DPN at the time of T2DM diagnosis, and five years after a type 1 diabetes diagnosis, with follow-up assessments conducted annually [17].

Diabetes mellitus, particularly type 2, is a chronic metabolic disorder with rising global prevalence, often leading to long-term complications, including neurological manifestations. The onset and severity of these complications are closely linked to the duration of the disease. However, limited local data exist comparing neurological outcomes across different durations of T2DM. Understanding this relationship is essential for early diagnosis, timely intervention, and prevention of debilitating complications. Therefore, this study was conducted to compare the neurological manifestations of T2DM with respect to the duration of diabetes.

## Materials and methods

This cross-sectional study was carried out at a secondary care hospital using a non-probability convenience sampling technique. Patients attending outpatient clinics were enrolled after obtaining written informed consent, and ethical approval was obtained from the Research Ethical Committee (REC) of 50-bedded Sindh Government Hospital, Karachi, with the reference number #SGH/(2A/Landhi)1479 dated 16-08-2024. The study duration was approximately six months, from October 1, 2024, to March 31, 2025. A total of 420 individuals with T2DM, aged between 40 and 65 years, were evenly divided into three groups of 140 based on the duration of their diabetes: Group A included those diagnosed within the past year, Group B consisted of individuals with one to five years of diabetes, and Group C comprised those with a disease duration exceeding five years. Individuals with type 1 diabetes mellitus (T1DM), hypoglycemia, a history of surgical procedures, or who had received chemotherapy were excluded from the study.

The duration of diabetes was determined through a combination of patient self-reported history and verification from medical records. Clinical evaluations were conducted to assess glycemic control and identify potential complications in individuals with T2DM. Glycemic control was primarily determined through measurements of glycosylated hemoglobin (HbA1c) and postprandial blood glucose levels. HbA1c provides an indicator of long-term glucose regulation, while postprandial glucose was measured two hours after a standard meal to evaluate metabolic response. A comprehensive assessment of diabetic complications was also undertaken, including cardiovascular evaluation through physical examination, blood pressure monitoring, and heart rate analysis. Demographic information such as age, gender, height, weight, physical activity levels, dietary patterns, and smoking habits was collected via structured questionnaires. Additional medical history was recorded, including sleep-related concerns like insomnia and abnormal sleep behaviors, along with heart rate and random blood sugar (RBS) readings. Peripheral neuropathy was assessed based on the presence and intensity of symptoms such as tingling or numbness in the extremities, burning sensations in the legs or feet, touch hypersensitivity, muscle cramps or pain, and symptom exacerbation during nighttime. Autonomic neuropathy symptoms were evaluated through indicators such as loss of appetite, insomnia, excessive thirst or hunger, fatigue, fruity breath odor, and cold sweats. Central nervous system involvement was identified by assessing mood disturbances, irritability, confusion, and difficulties with concentration.

Data was analyzed using IBM SPSS Statistics for Windows, Version 20 (Released 2012; IBM Corp., Armonk, New York, United States). Socio-demographic characteristics and neurological symptoms associated with T2DM were summarized using frequencies and percentages. Quantitative variables were presented as means along with standard deviations. To assess the association between neurological symptoms and T2DM, the chi-square test was applied. Furthermore, the Kruskal-Wallis test was utilized to evaluate differences in the means of demographic variables. A p-value of <0.05 was considered statistically significant.

## Results

The study included 420 patients with T2DM based on the duration of diabetes. The mean age of patients in Group A was significantly higher (60.27 ± 15.81 years) compared to Group B (52.47 ± 14.35 years) and Group C (54.65 ± 14.39 years) (p < 0.001). BMI was significantly different across the groups (p = 0.017), with Group A having the highest BMI (26.21 ± 11.31 kg/m^2^), followed by Group B (25.85 ± 9.85 kg/m^2^) and Group C (23.77 ± 11.54 kg/m^2^). Respiratory rate showed significant variation among the groups (p < 0.001), with Group A recording the highest mean value (20.37 ± 5.92 cycles/min), followed by Group C (19.86 ± 5.80 cycles/min) and Group B (17.3 ± 5.10 cycles/min). Heart rate was also significantly different (p < 0.001), with Groups A and C having similar mean values, while Group B had a lower mean heart rate. Blood pressure values did not differ significantly across the groups (p = 0.126). However, the duration of blood pressure was significantly different among the groups (p = 0.009), with the longest mean duration observed in Group A (5.73 ± 5.83 years). RBS levels varied significantly across the groups (p < 0.001). Group A had the highest mean RBS (335.34 ± 96.42 mg/dL), followed by Group B (328.3 ± 110.95 mg/dL), whereas Group C had the lowest RBS levels (227.89 ± 89.4 mg/dL), as presented in Table 1.

**Table 1:** Demographic details, clinical characteristics, and physiological parameters of patients with type 2 diabetes mellitus with respect to duration of diabetes. Data were expressed as mean ± standard deviation (SD). Kruskal-Wallis test was applied to compare variables across the groups. A p-value of < 0.05 was considered statistically significant.

Variables	Duration of Diabetes				
	Group A (Mean ± SD)	Group B (Mean ± SD)	Group C (Mean ± SD)	H-value	p-value
Age (years)	60.27 ± 15.81	52.47 ± 14.35	54.65 ± 14.39	19.806	<0.001
BMI (kg/m^2^)	26.21 ± 11.31	25.85 ± 9.85	23.77 ± 11.54	8.163	0.017
Respiratory rate (cycles/min)	20.37 ± 5.92	17.3 ± 5.10	19.86 ± 5.80	22.570	<0.001
Blood pressure (mmHg)	179.57 ± 43.99	172.53 ± 47.02	176.0 ± 50.55	4.144	0.126
Duration of blood pressure (years)	5.73 ± 5.83	4.17 ± 4.05	4.88 ± 3.24	9.315	0.009
Heart rate (beats/min)	87.75 ± 10.45	80.1 ± 11.45	87.29 ± 11.4	35.044	<0.001
Random blood sugar (RBS) (mg/dL)	335.34 ± 96.42	328.3 ± 110.95	227.89 ± 89.4	106.077	<0.001

A significant association was observed between gender and the duration of diabetes (p < 0.001). In Group A, males were predominant (90, 64.3%) compared to females (50, 35.7%), whereas Group B had a higher proportion of females (104, 74.3%) compared to males (36, 25.7%). In Group C, males were more prevalent (85, 59.3%) than females (57, 40.7%). The socioeconomic status of the participants varied significantly across the groups (p = 0.002). The proportion of participants from a high socioeconomic background decreased with increasing duration of diabetes. A significant association was found between dyslipidemia and diabetes duration (p < 0.001). However, no significant association was observed between hypertension and diabetes duration (p = 0.926), with similar prevalence rates across the groups. A significant difference was noted in smoking history among the groups (p < 0.001). Group A had the highest proportion of smokers (66, 47.1%), while Group B had the lowest (21, 15.0%). In Group C, 42 (30.0%) participants were smokers. A highly significant association was found between the type of diabetes therapy and the duration of diabetes (p < 0.001), as presented in Table 2.

**Table 2:** Comparison of demographic, lifestyle, and clinical characteristics with respect to duration of diabetes. Categorical variables were presented as frequencies with percentages (n (%)). The Pearson chi-square test was applied to assess associations between variables across the groups. A p-value of < 0.05 was considered statistically significant.

Variables		Duration of Diabetes				
		Group A, n (%)	Group B, n (%)	Group C, n (%)	Pearson chi-square	p-value
Gender	Male	90 (64.3)	36 (25.7)	83 (59.3)	49.277	<0.001
	Female	50 (35.7)	104 (74.3)	57 (40.7)		
Socioeconomic status	Low	22 (15.7)	27 (19.3)	20 (14.3)	16.767	0.002
	Middle	70 (50.0)	84 (60.0)	98 (70.0)		
	High	48 (34.3)	29 (20.7)	22 (15.7)		
History of dyslipidemia	Yes	106 (75.7)	116 (82.9)	83 (59.3)	20.572	<0.001
	No	34 (24.3)	24 (17.1)	57 (40.7)		
History of hypertension	Yes	97 (69.3)	96 (68.6)	94 (67.1)	0.154	0.926
	No	43 (30.7)	44 (31.4)	46 (32.9)		
History of smoking	Yes	66 (47.1)	21 (15.0)	42 (30.0)	34.035	<0.001
	No	74 (52.9)	119 (85.0)	98 (70.0)		
Therapy used	Oral hypoglycemic drugs	65 (46.4)	64 (45.7)	34 (24.3)	44.564	<0.001
	Insulin	44 (31.4)	30 (21.4)	30 (21.4)		
	Diet only	8 (5.7)	11 (7.9)	31 (22.1)		
	Diet along with oral hypoglycemic drugs	15 (10.7)	16 (11.4)	29 (20.7)		
	Diet along with insulin	8 (5.7)	19 (13.6)	16 (11.4)		

Significant differences in peripheral neuropathy symptoms were observed across the three groups. Tingling or numbness in the hands or feet was most common in Group C (114, 81.4%), followed by Group A (86, 61.4%) and Group B (68, 48.6%) (p < 0.001). Burning pain in the legs or feet was most frequently reported in Group B, followed by Groups A and C (p = 0.001). Sensitivity to touch was highest in Group B compared to Groups A and C (p = 0.013). Muscular pain or cramps were highly prevalent in Groups B (134, 95.7%) and C (133, 95.0%) compared to Group A (117, 83.6%) (p < 0.001). Symptoms worsening at night were more commonly reported in Groups A and B, but significantly less in Group C (p < 0.001). Among autonomic neuropathy symptoms, increased thirst was significantly more common in Group B than in Groups A and C (p < 0.001). Fatigue was most prevalent in Group B (133, 95.0%), followed by Group A (117, 83.6%) and Group C (111, 79.3%) (p < 0.001). Sweet-smelling breath was significantly more frequent in Groups A (81, 57.9%) and B (79, 56.4%) than in Group C (27, 19.3%) (p < 0.001). Cold sweating was also highest in Group B (107, 76.4%), followed by Group C (100, 71.4%) and Group A (80, 57.1%) (p = 0.002). Finally, irritability or mood changes were more common in Group B, compared to Groups C and A (p = 0.002), as presented in Table 3.

**Table 3:** Neurological symptoms in patients with type 2 diabetes with respect to duration of diabetes. Categorical variables were presented as frequencies with percentages (n (%)). The Pearson chi-square test was applied to assess associations between variables across the groups. A p-value of < 0.05 was considered statistically significant.

Variable			Duration of Diabetes				
			Group A, n (%)	Group B, n (%)	Group C, n (%)	Pearson chi-square	p-value
Peripheral neuropathy symptoms	Tingling or numbness in the hands or feet	Yes	86 (61.4)	68 (48.6)	114 (81.4)	33.240	<0.001
		No	54 (38.6)	72 (51.4)	26 (18.6)		
	Burning pain in your legs or feet	Yes	77 (55.0)	97 (69.3)	67 (47.9)	13.630	0.001
		No	63 (45.0)	43 (30.7)	73 (52.1)		
	Too sensitive feet on touch	Yes	38 (27.1)	51 (36.4)	29 (20.7)	8.651	0.013
		No	102 (72.9)	89 (63.6)	111 (79.3)		
	Muscular pain or cramps in legs or feet	Yes	117 (83.6)	134 (95.7)	133 (95.0)	16.589	<0.001
		No	23 (16.4)	6 (4.3)	7 (5.0)		
	Symptoms worse at night	Yes	79 (56.4)	76 (54.3)	45 (32.1)	20.294	<0.001
		No	61 (43.6)	64 (45.7)	95 (67.9)		
Autonomic neuropathy symptoms	Loss of appetite	Yes	89 (63.6)	84 (60.0)	80 (57.1)	1.213	0.545
		No	51 (36.4)	56 (40.0)	60 (42.9)		
	Insomnia	Yes	68 (48.6)	62 (44.3)	62 (44.3)	0.691	0.708
		No	72 (51.4)	78 (55.7)	78 (55.7)		
	Increased thirst	Yes	53 (37.9)	85 (60.7)	45 (32.1)	26.030	<0.001
		No	87 (62.1)	55 (39.3)	95 (67.9)		
	Fatigue	Yes	117 (83.6)	133 (95.0)	111 (79.3)	15.302	<0.001
		No	23 (16.4)	7 (5.0)	29 (20.7)		
	Increased hunger	Yes	34 (24.3)	41 (29.3)	24 (17.1)	5.789	0.055
		No	106 (75.7)	99 (70.7)	116 (82.9)		
	Sweet-smelling breath	Yes	81 (57.9)	79 (56.4)	27 (19.3)	54.212	<0.001
		No	59 (42.1)	61 (43.6)	113 (80.7)		
	Cold sweating	Yes	80 (57.1)	107 (76.4)	100 (71.4)	12.962	0.002
		No	60 (42.9)	33 (23.6)	40 (28.6)		
Central nervous system symptoms	Irritability or having other mood changes	Yes	100 (71.4)	123 (87.9)	104 (74.3)	12.513	0.002
		No	40 (28.6)	17 (12.1)	36 (25.7)		
	Confusion or difficulty concentrating	Yes	61 (43.6)	57 (40.7)	43 (30.7)	5.399	0.067
		No	79 (56.4)	83 (59.3)	97 (69.3)		

## Discussion

Diabetic neuropathy is a serious complication of diabetes, influenced by various risk factors. This study highlighted the prevalence of neurological symptoms in individuals with T2DM, examining the relationship between symptom occurrence and disease duration.

In the present study, a significant association was found between the duration of T2DM and various demographic and clinical variables, including age, and specific autonomic neuropathy symptoms. Notably, patients in Group A (newly diagnosed, less than one year) had a significantly higher mean age compared to those in Groups B and C. The present study did not find a significant difference in insomnia or loss of appetite across groups but did reveal that fatigue and increased thirst were significantly more prevalent in patients with intermediate disease duration (one to five years), which contrasts with a previous cross-sectional study where the average age of diabetic patients (with more than one year history) was lower (42.8 ± 14.4 years) and the mean disease duration was longer (7.7 ± 7.2 years). This earlier study reported a direct and significant relationship between longer disease duration and poorer general health, including physical symptoms, anxiety, insomnia, and depression (p < 0.001) [20].

Similarly, another study found that age and age at diagnosis were independently associated with macrovascular events and death, but not with microvascular complications, except when interacting with diabetes duration. Specifically, the effect of duration on microvascular risk was more pronounced in younger patients, suggesting an age-dependent vulnerability. Likewise, it was reported that longer diabetes duration significantly increased the risk of macrovascular events, microvascular events, and all-cause mortality, indicating a progressive impact of the disease on systemic health [21]. In the present study, longer diabetes duration was associated with a higher prevalence of neuropathic symptoms such as tingling, numbness, and muscle cramps, while metabolic parameters such as BMI and RBS were highest in those with a shorter duration of diabetes.

In another study, patients with neuropathy had a longer mean diabetes duration and higher HbA1c levels, reinforcing the notion that prolonged exposure to hyperglycemia contributes to nerve damage [22]. The findings of the present study align with those of the case-control study conducted in Pakistan, particularly in highlighting the significant association between the duration of T2DM and the presence of diabetic neuropathy. In the present study, symptoms of peripheral neuropathy such as tingling, numbness, muscle cramps, and burning pain were more prevalent in patients with a longer duration of diabetes, particularly in Group C (more than five years). Additionally, autonomic symptoms such as fatigue, cold sweating, and irritability were also significantly more common in patients with longer-standing diabetes.

A study by Oguejiofor et al. reported a lower incidence of polyneuropathy in patients with a diabetes duration of less than five years and a marked increase in those with more than 15 years of the disease [23]. Similarly, the UK study reported neuropathy in 36% of individuals with over 10 years of diabetes, compared to only 20% in those with a five-year duration [24]. The findings of the present study were consistent with those reported by Oguejiofor et al., and the large UK-based study showed that peripheral neuropathy symptoms such as tingling, numbness, muscle cramps, and burning pain were significantly more common in patients with longer diabetes duration, particularly those in Group C (more than five years). It also demonstrated a clear trend of increasing neuropathic symptoms with longer disease duration.

Likewise, another study examined the prevalence of DPN and its associated risk factors in patients with T1DM and T2DM. The overall DPN prevalence in the cohort was 40.3%, with 29.1% in T1D patients and 42.2% in T2D patients (p < 0.005). The prevalence of DPN increased with age, starting at 11.9% in individuals ≤40 years and rising to over 50% in those aged >70 years (p < 0.001). In terms of diabetes duration, T2D patients diagnosed with the condition had a high DPN prevalence of 35%. The prevalence significantly increased 5 and 25 years after T1D diagnosis and 25 years after T2D diagnosis (p < 0.001) [25]. The overall prevalence of neuropathy in this study was slightly higher than previously reported (~30%), with a rise in prevalence observed with both age and duration of diabetes [19], which aligns with findings from other population-based studies [26]. A study of 258 youth with T2D (mean age 22 ± 4 years) found a higher prevalence of 19% after 5-10 years of diabetes duration, increasing to 36% after more than 10 years [26]. The present study was consistent with the above-cited studies and showed that patients with a longer duration of diabetes exhibited higher prevalence rates of peripheral neuropathy symptoms, including tingling or numbness, muscular pain, and cramps, indicating progressive nerve damage over time.

A study from Saudi Arabia involving 285 participants (58.9% with T2DM and 41.1% with T1DM) reported that 71.9% were on insulin therapy. Neuropathic pain was significantly more prevalent among T2DM patients (66.5%, p < 0.001). Female patients experienced a higher frequency of neuropathic symptoms, including foot tenderness (40.8%), persistent burning pain (26.2% vs. 2.5% in men), and numbness or reduced sensation (19.4% vs. 1.3% in men). Moreover, 30.4% of women reported ongoing prickling or burning sensations compared to 8.9% of men, highlighting a significant gender disparity in symptom presentation (p < 0.001) [27]. As far as the present study is concerned, patients with a longer duration of diabetes showed a higher prevalence of peripheral neuropathy symptoms such as tingling, numbness, muscle pain, and cramps, indicating worsening nerve damage over time. Autonomic neuropathy symptoms, including increased thirst, fatigue, sweet-smelling breath, and cold sweats, were more commonly observed in those with an intermediate duration of diabetes, suggesting varying patterns of metabolic and autonomic dysfunction as the disease advances.

Previous epidemiological research has identified prolonged diabetes duration and elevated blood glucose levels as key risk factors for developing DPN [28]. Traditional cardiovascular risk factors, such as hypertension, smoking, dyslipidemia, and increased BMI, have also been linked to diabetes-related complications, including neuropathy [29]. However, contrasting evidence from another study pointed to depressive symptoms as a significant emerging risk factor for DPN, suggesting that individuals with T2DM who experience depression are at greater risk of neuropathy, regardless of demographic variables, disease duration, HbA1c levels, blood pressure, BMI, or cardiovascular risk [30]. These findings align with the present study, which demonstrated that comorbidities such as dyslipidemia, longer diabetes duration, lower socioeconomic status, and smoking had a statistically significant impact on the majority of neurological manifestations in T2DM patients (p < 0.001).

This study is limited by its cross-sectional design, which prevents establishing causal relationships. Additionally, self-reported symptoms may introduce recall bias, and factors such as medication adherence and comorbidities were not extensively analyzed. Future research should include longitudinal studies to assess disease progression over time and explore interventions for early neuropathy prevention. Regular screening, lifestyle modifications, and personalized treatment strategies are recommended to improve diabetes management and patient outcomes.

## Conclusions

This study concluded that peripheral neuropathy symptoms, such as tingling, numbness, burning pain, and muscular cramps, were markedly more prevalent among individuals with longer-standing diabetes (Group C), suggesting progressive nerve involvement over time. Interestingly, certain symptoms, such as burning pain, sensitivity to touch, and fatigue, were more pronounced in those with shorter disease duration (Group B), possibly reflecting early inflammatory or metabolic changes before overt nerve degeneration. Autonomic neuropathy symptoms, including increased thirst, fatigue, sweet-smelling breath, and cold sweating, also showed significant variation across groups, further emphasizing the complex and evolving nature of neurological involvement in T2DM.
